# Effect of Fruit Juice on Cholesterol and Blood Pressure in Adults: A Meta-Analysis of 19 Randomized Controlled Trials

**DOI:** 10.1371/journal.pone.0061420

**Published:** 2013-04-24

**Authors:** Kai Liu, Anhui Xing, Ka Chen, Bin Wang, Rui Zhou, Shihui Chen, Hongxia Xu, Mantian Mi

**Affiliations:** 1 Research Center for Nutrition and Food Safety, Institute of Military Preventive Medicine, Third Military Medical University, Chongqing Key Laboratory of Nutrition and Food Safety, Chongqing Medical Nutrition Research Center, Chongqing, P. R. China; 2 Center for Disease Control and Prevention of Shenyang Command, Shenyang, P. R. China; Aga Khan University, Pakistan

## Abstract

**Background:**

The effect of fruit juice on serum cholesterol and blood pressure in humans has generated inconsistent results. We aimed to quantitatively evaluate the effect of fruit juice on serum cholesterol and blood pressure in adults.

**Methods:**

We performed a strategic literature search of PubMed, EMBASE, and the Cochrane Library (updated to October, 2012) for randomized controlled trials that evaluated the effects of fruit juice on serum cholesterol and blood pressure. Study quality was assessed by using the Jadad scale. Weighted mean differences were calculated for net changes in cholesterol and blood pressure by using fixed-effects model. Prespecified subgroup and sensitivity analyses were conducted to explore the potential heterogeneity.

**Results:**

Nineteen trials comprising a total of 618 subjects were included in this meta-analysis. Fruit juice consumption borderlinely reduced the diastolic blood pressure (DBP) by 2.07 mm Hg (95% CI: −3.75, −0.39 mm Hg; p = 0.02), but did not show significant effects on total cholesterol (TC), high-density lipoprotein-cholesterol (HDL-C), low-density lipoprotein-cholesterol (LDL-C) concentrations or systolic blood pressure (SBP) values. A significant reduction of TC concentration was observed in low-median intake of total polyphenols group. Subgroup analyses for HDL-C and LDL-C concentrations did not show statistically significant results. No significant heterogeneity was detected for all the measures.

**Conclusion:**

This meta-analysis suggested that fruit juice had a borderline significant effect on reducing DBP, but had no effect on TC, HDL-C, LDL-C concentrations or SBP.

## Introduction

Cardiovascular disease (CVD) is one of the most common public health challenges to all nations. Although the mortality data from 1998 to 2008 showed that the death rate attributable to CVD declined by 30.6%, more than 2,200 Americans die of CVD each day, an average of 1 death every 39 seconds [1]. Epidemiologic studies have revealed that elevated concentrations of serum total cholesterol (TC) and low-density lipoprotein cholesterol (LDL-C) are independent risk factors for CVD [2], [3]. In addition, accumulating evidence suggests that hypertension is a major risk factor for CVD, such as heart failure, stroke, and myocardial infarction [4]. Therefore, the effective control on lipid metabolism and blood pressure will be greatly beneficial to CVD prevention.

It has been suggested that a diet rich in fruits and vegetables is associated with protection against CVD [5]. A recent meta-analysis showed that fruit consumption was negatively associated with the risk of coronary heart disease, and the finding indicated that the risk of coronary heart disease was decreased by 7% for each additional portion of daily fruit intake [6]. Fruit juices are generally less desirable than whole fruits since they contain less fiber. However, both of them contain the equivalent amount of other important and beneficial nutrients, such as polyphenols, antioxidants and folate with the whole fruits [7]. Previous human clinical trials investigating the effects of fruit juice on serum cholesterol and blood pressure have generated inconsistent results, and their sample sizes were relatively modest. Therefore, we conducted a meta-analysis of all published randomized controlled trials (RCTs) to quantitatively assess the effect of fruit juice on serum cholesterol and blood pressure.

## Methods

### Search Strategy

PubMed (updated to October 2012; http://www.ncbi.nlm.nih.gov/pubmed/), Embase (1980 to October 2012; http://www.embase.com/), the Cochrane Library (1985 to October 2012; http://www.cochrane.org/) database, and reference lists and reviews were searched for RCTs evaluating the effects of fruit juice on serum cholesterol and blood pressure in humans. The structured search strategies were performed using the keyword juice or juices. The search was restricted to the reports of clinical trials conducted in human subjects.

### Study Selection

Studies were selected for analysis if they met the following criteria: 1) subjects consumed fruit juice for ≥2 wk; 2) the study was an RCT conducted in human subjects with either a parallel or crossover design; 3) the baseline and endpoint values or their difference of serum TC, high-density lipoprotein-cholesterol (HDL-C), LDL-C concentrations, diastolic blood pressure (DBP) or systolic blood pressure (SBP) with SD or SEM or 95%CI were available for each group in the study; 4) fruit juice was not given as part of a multi-component supplement in the study; and 5) the study used a concurrent control group for the fruit juice treatment group and the difference between the control and treatment group was fruit juice consumption.

### Data Extraction and Quality Assessment

Data was collected onto a pre-piloted data extraction form which included the following creiteria: 1) study characteristics (authors, publication year, sample size, study design, population information, study duration, total polyphenols dose, type of intervention and type of diet); 2) net changes in serum cholesterol, and 3) mean changes in blood pressure. All values were converted to mg/dL for cholesterol by using the conversion factors 1 mg/dL = 0.0259 mmol/L [8]. If the outcomes were reported several times in different stages of trials, only values representing the final outcome concentrations at the end of trials were extracted for our meta-analysis.

Studies evaluated for potential inclusion in the meta-analysis were estimated for quality using the following criteria: 1) randomization; 2) double blinding (participant masking and researcher masking); 3) withdrawal reporting (total number and reasons for withdrawal); 4) allocation concealment; and 5) generation and use of random numbers. RCTs scored one point for each above areas addressed in the study design to earn a possible score of 0 (lowest quality) to 5 (highest quality) [9]. Studies receiving a score ≥4 were deemed to be of high quality whereas those receiving a score of <4 were considered lower quality.

### Statistical Analysis

Our meta-analysis was performed using STATA (Version 11; StataCorp, College Station, TX). Treatment effects were defined as weighted mean difference and 95% CIs calculated for net changes in serum cholesterol and blood pressure values. The statistic heterogeneity was assessed by using Cochran’s test (p<0.1). The I^2^ statistic was also calculated, and I^2^>50% was considered as significant heterogeneity across studies [10]. A random-effects model was used if significant heterogeneity was shown among the trials. Otherwise, results were obtained from a fixed-effects model.

Percent change in mean and SD values were excluded when extracting SD values for an outcome. SD values were calculated from standard errors, 95% CIs, p-values, or t if they were not available directly. Additionally, change-from-baseline SD values were imputed as suggested by Follmann et al [11], assuming a correlation coefficient of 0.5.

Publication bias was assessed using funnel plots and the Egger’s regression test. Previously defined subgroup analyses were performed to examine the possible sources of heterogeneity within these studies and included health status, study design, type of intervention, duration, total polyphenols dose, and Jadad score. Additional sensitivity analyses were also performed according to the Handbook for Systematic Review of Interventions of Cochrane software (Version 5.0.2; The Cochrane Collaboration, Oxford, United Kingdom).

## Results

### Results of Literature Search

**Figure 1:**
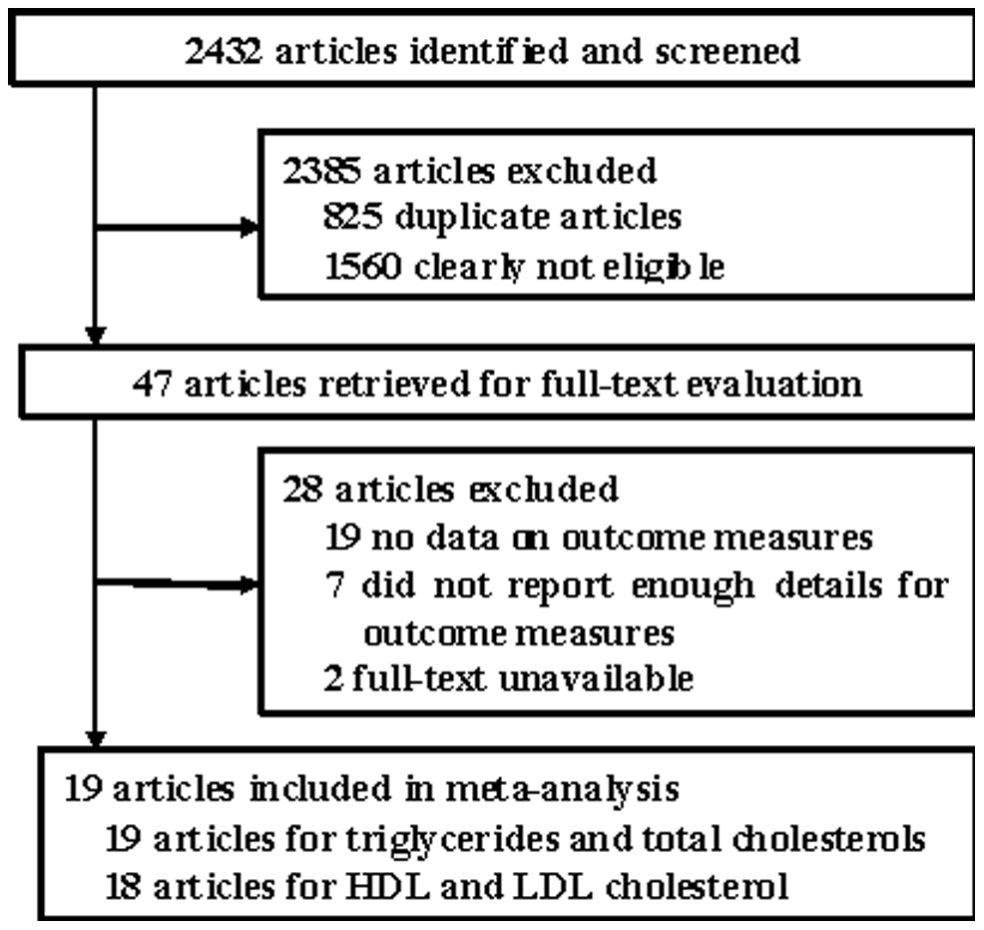
Flow diagram showing the number of citations retrieved by individual searches of articles included in the review.

Detailed processes of the relevant study selection are shown in **Figure 1**. A total of 2432 reports were initially identified, and 2385 articles were excluded either because of duplication or because they were clearly not relevant to the current meta-analysis after a review of titles and abstracts. Thus, 47 articles remained for more detailed examination. Among them, an additional 28 articles were excluded for the following reasons: 1) 19 articles were excluded because there was no data on outcome measures; 2) 7 articles were discarded because they did not report SD or baseline or endpoint or mean difference for primary or secondary outcome measures; and 3) full-texts of two studies were unavailable. Ultimately, 19 articles were selected for inclusion in the meta-analysis [12]–30.

### Study Characteristics

The characteristics of for each study included in the meta-analysis are provided in **Table 1** **.** A total of 618 subjects included in this meta-analysis, and subject numbers for each study ranged from 12 to 63. The total amount of polyphenols contained in fruit juice ranged from 65.2 to 2660 mg/d (median: 927 mg/d). Study duration varied from 2 wk to 3 mo (median: 6 wk). Subjects in ten studies came from North American, 6 studies included subjects from Europe, and 3 studies selected subjects from Asia. Of the 19 trials included in the current analysis, 12 trials used fruit juice with multi-nutrients (polyphenols, vitamins, and sugar et al.), and seven studies ruled out the vitamins and sugar interferences in the effect of polyphenols on blood cholesterol concentrations. Most studies (15 of 19) used the parallel design. Of the 18 studies which suggested their participants maintain the usual diet, 11 studies limited intake of fruit, vegetable, fruit juice or other source of polyphenols, and one study recommended that subjects maintain physical exercise during the study.

**Table 1:** Characteristics of 19 randomized controlled trials included in analysis.

Study	No. of subjects	Country	Study design	Participants	Duration	Juice group (Total polyphenols)	Control group	Type of diet
**Murkovic 2004b**	34	Austrilia	Parallel	Healthy	2 wk	400 mg elderberry juice powder (400 mg/d)	Placebo	Usual diet
**Reshef 2005a**	12	Israel	Crossover	Stage I hypertension	5 wk	500 ml sweetie fruit juice (444.5 mg/d)	Placebo (115 mg/d)	Usual diet
**Summer 2005b**	39	US	Parallel	Coronary heart disease	3 mo	240 ml pomegranate juice (NR)	Placebo	NR
**Castilla 2006b**	38	Spain	Parallel	Hemodialysis	14 d	100 ml concentrated red grape juice (640 mg/d)	No intervention	Usual diet, avoid intake of fruit and vegetables
**Bannni 2006b**	23	US	Parallel	Type 2 diabetes mellitus	28 d	150 ml muscadine grape juice (NR)	No intervention	Usual diet
**Cerda 2006a**	30	Spain	Parallel	Chronic obstructive pulmonary disease	5 wk	400 ml pomeranate juice (2660 mg/d)	Placebo	Controlled diet, limit berries, pomegranates, chocolate, nuts and wine
**Duthie 2006b**	20	Scotland	Parallel	Healthy	2 wk	750 ml cranberry juice (852 mg/d)	Placebo (6.72 mg/d)	Usual diet
**Castilla 2008b**	16	Spain	Parallel	Hemodialysis	2 wk	100 ml concentrated red grape juice (640 mg/d)	No intervention	Usual diet, avoid intake of fruit and vegetables
**Park 2009a**	40	Japan	Parallel	Borderline isolate hypertension	8 wk	418 ml grape juice (885 mg/d)	Placebo	Usual diet
**Hollis 2010a**	51	US	Parallel	Overweight	12 wk	480 ml concord grape juice (933.6 mg/d)	Placebo	Usual diet, avoid intake of other juices
**Basu 2010b**	27	US	Parallel	Metabolic syndrome	8 wk	50 g freeze-dried strawberry juice (2160 mg/d)	Water	Usual diet, avoid intake of other berries
**Basu 2010b**	48	US	Parallel	Metabolic syndrome	8 wk	50 g freeze-dried blueberry juice (1624 mg/d)	Water	Usual diet, avoid intake of other berries, green tea, cocoa, and soy
**Karlsen 2010b**	62	Norway	Parallel	Cardiovascular disease risk	4 wk	330 ml bilberry (NR)	Water	Usual diet, avoid intake of other berries and berry products
**Dohadwala 2010a**	63	US	Crossover	Stage I hypertension	8 wk	490 ml concord grape juice (965 mg/d)	Placebo	Usual diet, avoid intake of grape juice, wine, grape products, green or black tea, dark juices
**Aptekmann 2010b**	26	Brazil	Parallel	Overweight	3 mo	500 ml concentrated orange juice (65.2 mg/d)	No intervention	Usual diet, aerobic training 3 times per week
**Gonzalez-Ortiz 2011b**	20	US	Parallel	Obesity	1 mo	120 ml pomegranate juice (NR)	Placebo	Usual diet
**Dohadwala 2011a**	44	US	Crossover	Coronary artery disease	4 wk	480 ml cranberry juice (835 mg/d)	Placebo	Usual diet, avoid intake of grape juice, wine, grape products, green or black tea, dark juices
**Morand 2011b**	24	France	Crossover	Overweight	4 wk	500 ml orange juice (341.9 mg/d)	Control drink	Usual diet, avoid intake of citrus-containing foods and limit tea, coffee, cocoa, wine, fruit juice ≤200 mL/d
**Basu 2011a**	31	US	Parallel	Metabolic syndrome	8 wk	240 ml low-energy cranberry juice (458 mg/d)	Placebo	Usual diet, avoid intake of berries, green tea, cocoa, and soy products

The studies by Reshef (2005), Cerda (2006), Park (2009), Hollis (2010), Dohadwala (2010), Dohadwala (2011), and Basu (2011) used fruit juice with polyphenols as main nutrients, and the study by Murkovic (2004), Summer (2005), Castilla (2008), Bannni (2006), Duthie (2006), Castilla (2008), Basu (2010), Karlsen (2010), Aptekmann (2010), Gonzalez-Ortiz (2011), and Morand (2011) used fruit juice with multi-nutrients (polyphenols, vitamins, and sugar et al.); a usual diet was similar to a conventional diet.

NR, not reported.

### Data Quality

Study quality was assessed by the Jadad scale [8], and the results were varied. Seven trials [14], [17], [21], [25], [24], [29], [30] were classified as high quality (Jadad score ≥4) and the remaining 12 trials were low quality (Jadad score <4). All 7 high-quality trials had adequate allocation concealment (i.e., conducted by a third-party manufacturer or using opaque envelopes) and 2 high-quality trials reported the generation of random numbers or randomization list. Details of dropouts were reported in 17 trials [10]–[16], [18]–[27].

### Effect of Fruit Juice on Serum Cholesterol and Blood Pressure

**Figure 2:**
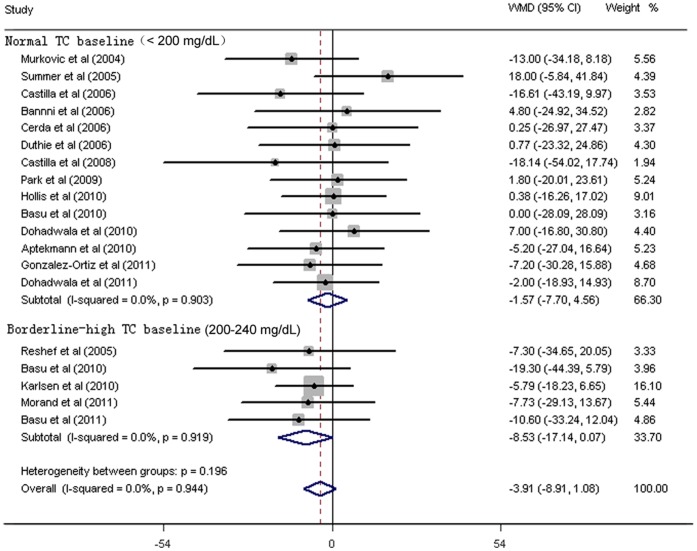
Meta-analysis of effects of fruit juice on total cholesterol (TC) concentrations. The result was obtained from a fixed-effects model. Sizes of data markers indicate the weight of each study in this analysis. The basis of classification of TC is referred to the National Cholesterol Education Program guidelines, Adult Treatment Panel III. WMD, weighted mean difference.

**Figure 3:**
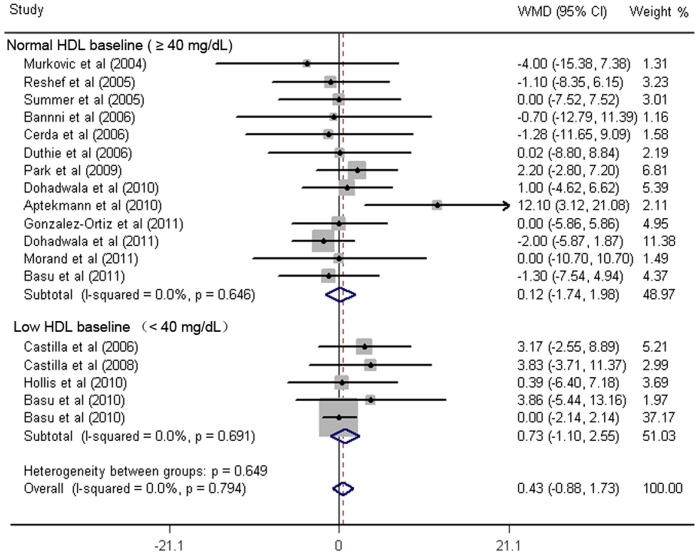
Meta-analysis of effects of fruit juice on high-density lipoprotein-cholesterol (HDL-C) concentrations. The result was obtained from a fixed-effects model. Sizes of data markers indicate the weight of each study in this analysis. The basis of classification of HDL-C is referred to the National Cholesterol Education Program guidelines, Adult Treatment Panel III. WMD, weighted mean difference.

**Figure 4:**
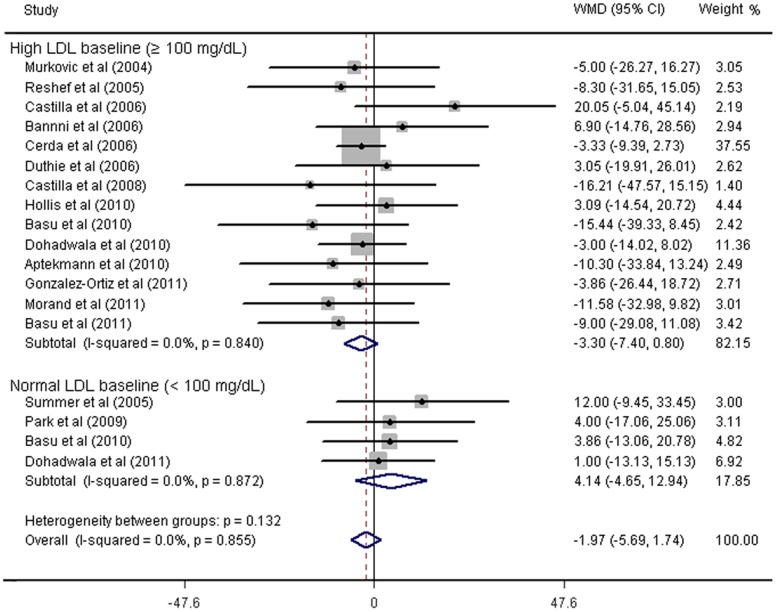
Meta-analysis of effects of fruit juice on low-density lipoprotein-cholesterol (LDL-C) concentrations. The result was obtained from a fixed-effects model. Sizes of data markers indicate the weight of each study in this analysis. The basis of classification of LDL-C is referred to the National Cholesterol Education Program guidelines, Adult Treatment Panel III. WMD, weighted mean difference.

**Figure 5:**
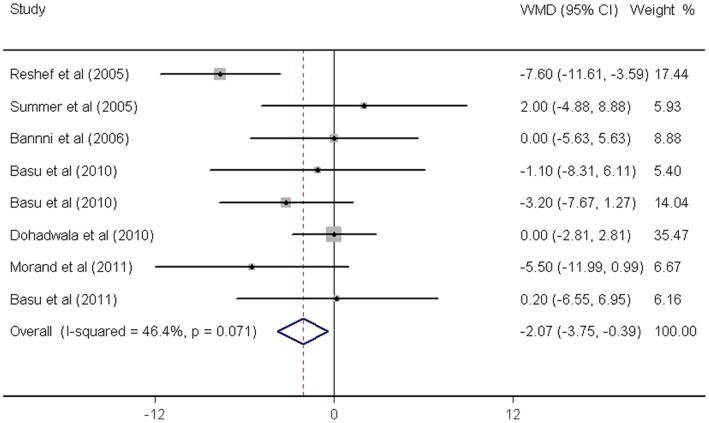
Meta-analysis of effects of fruit juice on diastolic blood pressure (DBP). The result was obtained from a fixed-effects model. Sizes of data markers indicate the weight of each study in this analysis. WMD, weighted mean difference.

**Figure 6:**
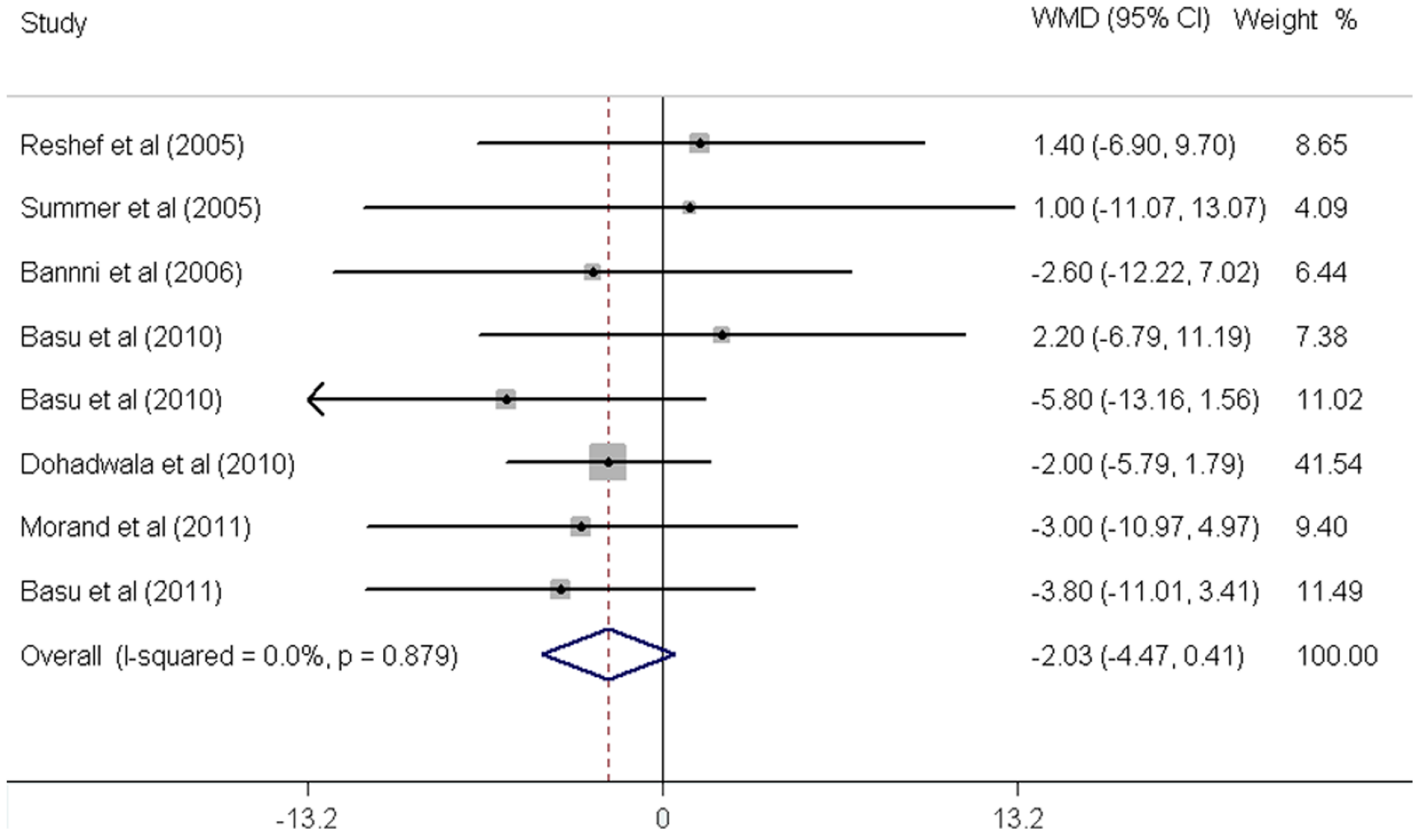
Meta-analysis of effects of fruit juice on systolic blood pressure (SBP). The result was obtained from a fixed-effects model. Sizes of data markers indicate the weight of each study in this analysis. WMD, weighted mean difference.

As shown in **Figure 2** **–** **6** **,** fruit juice significantly lowered DBP, but did not significantly affect TC, HDL-C, LDL-C concentrations or SBP values. No significant heterogeneity was found for any of the outcome measures, and the results were reported on the basis of fixed-effects models. For the 19 trials that reported data on TC concentrations, no significant mean differences were observed in subjects supplemented with fruit juice (−3.91 mg/dL 95% CI: −8.91, 1.08 mg/dL, p = 0.12; **Figure 2**) compared with control subjects. The mean difference change in HDL-C concentrations were reported in 19 trials and no significant difference was found (0.43 mg/dL 95% CI: −0.88, 6.18 mg/dL, p = 0.52; **Figure 3**). LDL-C concentrations were measured in 19 trials and the pooled estimated net change was −1.97 mg/dL (95% CI: −5.69, 1.74 mg/dL; p = 0.90; **Figure 4**). The mean difference change in DBP values was reported in 8 studies, which was significantly decreased by 2.07 mm Hg (95% CI: −3.75, −0.39 mm Hg; p = 0.02; **Figure 5**). In addition, 8 studies examined SBP values, and no significant mean difference change was observed (**Figure 6**).

### Sensitivity and Subgroup Analysis

Subgroup analyses showed that the pooled effects of fruit juice were not influenced by TC baseline concentrations, nutrient constitution of fruit juice, study design, type of intervention, study duration, or study region. A significant reduction of TC was detected in low-median total polyphenols group when we stratified studies according to the dose of total polyphenols. Subgroup analyses for the effect of fruit juice on HDL-C and LDL-C concentrations were not significantly different from the overall net change of HDL-C and LDL-C concentrations. Results are summarized in Figure 2–4 and **Table 2**. The results of sensitivity analysis showed that the pooled effects of fruit juice on serum cholesterol concentrations were not altered when analyses were limited to high-quality studies and were not changed after imputation using a correlation coefficient of 0.5. In addition, we found no significant change of outcome measures through systematic removal of each trial during sensitivity analysis. Overall, no significant heterogeneity was found for TC, HDL-C and LDL-C concentrations, and the results were calculated on the basis of fixed-effects models.

**Table 2:** Subgroup analyses of TC, HDL-C, and LDL-C stratified by previously defined study characteristics.

Study characteristics	Total cholesterol				HDL cholesterol				LDL cholesterol			
	No. of trials	Net change(95% CI)	p for heterogeneity	p	No. of trials	Net change(95% CI)	p for heterogeneity	p	No. of trials	Net change(95% CI)	p for heterogeneity	p
**Fruit juice nutrient constitution**												
** Multi-nutrients**	12	−5.61 (−12.00, 0.78)	0.76	0.08	11	0.87 (−0.77, 2.50)	0.51	0.30	11	−0.64 (−7.35, 6.07)	0.52	0.85
** Polyphenols (main nutrients)**	7	−3.92 (−9.26, 6.75)	0.96	0.76	7	−1.34 (−2.49, 1.83)	0.91	0.76	7	−2.57 (−7.03, 1.90)	0.95	0.26
**Study design**												
** Parallel**	15	−4.33 (−9.98, 1.32)	0.86	0.13	14	0.81 (−0.66, 2.28)	0.69	0.28	14	−1.50 (−5.76, 2.76)	0.72	0.49
** Crossover**	4	−2.42 (−13.10, 8.25)	0.81	0.66	4	−0.97 (−3.79, 1.84)	0.79	0.50	4	−3.49 (−11.10, 4.12)	0.78	0.37
**Type of intervention**												
** Berries juice**	7	−6.59 (−13.91, 0.72)	0.89	0.08	6	−0.45 (−2.15, 1.26)	0.84	0.61	6	−2.15 (−9.85, 5.56)	0.77	0.59
** Grapes juice**	6	−1.36 (−10.98, 8.26)	0.82	0.60	6	1.94 (−0.66, 4.53)	0.97	0.14	6	1.32 (−1.64, 8.68)	0.51	0.73
** Pomegranate juice**	3	3.71 (−10.45, 17.87)	0.32	0.61	3	−0.21 (−4.44, 4.01)	0.98	0.92	3	−2.30 (−7.95, 3.35)	0.40	0.43
** Orange juice**	2	−6.49 (−21.78, 8.80)	0.87	0.41	2	6.41 (−5.42, 18.25)	0.09	0.29	2	−11.0 (−26.84, 4.83)	0.94	0.17
**Duration**												
** <6 wk (low median)**	11	−6.01 (−12.47, 0.45)	0.99	0.07	10	−0.18 (−2.37, 2.01)	0.92	0.87	10	−2.31 (−6.92, 2.30)	0.73	0.33
** ≥6 wk (high median)**	8	−0.81 (−8.68, 7.06)	0.32	0.84	8	0.99 (−0.92, 2.91)	0.35	0.31	8	−1.34 (−7.62, 4.93)	0.81	0.68
**Total polyphenols dose**												
** <927 mg/d (low median)**	11	−6.37 (−12.60, −0.14)	0.99	0.045	10	0.76 (−1.27, 2.80)	0.27	0.46	10	−2.57 (−9.27, 4.13)	0.69	0.45
** ≥927 mg/d (high median)**	5	−1.73 (−11.95, 8.48)	0.65	0.74	5	0.25 (−1.60, 2.10)	0.94	0.79	5	−2.71 (−7.48, 2.06)	0.71	0.27
**Jadad score**												
** Low (2, 3)**	12	−6.08 (−12.97, 0.82)	0.96	0.08	12	0.64 (−0.94, 2.23)	0.39	0.43	12	−0.60 (−6.69, 5.49)	0.77	0.85
** High (≥4)**	7	−1.53 (−8.77, 5.71)	0.94	0.68	6	−0.04 (−3.00, 2.91)	0.79	0.98	6	−2.79 (−7.48, 1.90)	0.65	0.24
**Region**												
** North American**	10	−11.55 (−8.53, 5.43)	0.72	0.66	10	0.14 (−1.36, 1.65)	0.44	0.85	10	−0.96 (−6.53, 4.61)	0.81	0.74
** European**	6	−6.49 (−14.97, 1.99)	0.91	0.13	5	1.93 (−1.62, 5.49)	0.90	0.29	5	−2.80 (−8.23, 2.64)	0.31	0.31
** Asia**	3	−6.17 (−19.45, 7.12)	0.63	0.36	3	0.54 (−3.33, 4.41)	0.54	0.78	3	−2.74 (−15.34, 9.86)	0.72	0.67

### Publication Bias

Funnel plots and Egger’s tests indicated no significant publication bias in the current meta-analysis of TC, HDL-C, LDL-C, DBP and SBP values (Egger’s test: p = 0.86, 0.34, 0.72, 0.99, and 0.57 respectively).

## Discussion

Our meta-analysis showed that fruit juice supplementation significantly lowered DBP values but did not significantly affect TC, HDL-C, LDL-C concentrations or SBP values. Subgroup analyses showed that the pooled effects of fruit juice on TC concentrations were not influenced by TC baseline concentrations, health status, study design, type of intervention, or study duration. A significant reduction of TC was observed in the group with low-median total polyphenols intake when we stratified studies according to dose of total fruit juice polyphenols. Subgroup analyses for the effect of fruit juice on HDL-C and LDL-C concentrations did not show statistically significant outcomes. Changes in TC, HDL-C, and LDL-C concentrations remained non-significant when analyses were limited to high quality studies.

Although we did not find the significant association between the consumption of fruit juice and TC concentration, the subgroup analysis stratified by TC baseline concentrations suggested that fruit juice had a favorable effect on decreasing TC concentration in borderline-high TC baseline group (−8.53 mg/dL 95% CI: −17.14, 0.07 mg/dL, p* = *0.052) rather than in the normal TC baseline group. Interestingly, we also find more favorable trends in high LDL-C baseline and Low HDL-C baseline group than that in normal LDL-C baseline and normal HDL-C group, although it was not statistically significant. Evidence for subgroup analysis for borderline-high TC baseline and low HDL-C baseline group was limited due to the small number of studies, which might partly explain why we did not find any beneficial effects of fruit juice on TC or HDL-C concentrations. Another possibility is that fruit juice has less fiber than whole fruit, since previous meta-analysis conducted by Brown et al [31] had found that soluble fibers had significant effects on reducing TC and LDL-C concentrations. In our meta-analysis, it was unclear why fruit juice significantly reduced the TC concentrations in low-median total polyphenols group, but not in high-median total polyphenols group. The finding might partly due to the increased daily calories intake accompanied by the additional consumption of fruit juice, since most trials advised subjects to keep their usual diet during the study period. Therefore, it might be advisable to recommend that fruit juice should be incorporated into a diet, with no net increase in the total calory intake.

Consistent with the result of our study, a recently published RCT trial including 690 participants aged 25–64 years showed that the increased fruit and vegetable intake could significantly decrease DBP by 1.5 mm Hg. Overall outcome of DBP by our meta-analysis showed that intake of fruit juice statistically significantly reduced DBP value by 2.07 mm Hg. In addition, we also found a favorable trend of SBP decreasing in the intervention groups, although the mean difference change in SBP was borderline-significant. It is estimated that a reduction of 2 mm Hg in DBP results in a decrease of incidence rates of hypertension and coronary heart disease by approximately 17% and 6%, respectively [32]. A recent meta-analysis also suggested that the observed differences of 10 to 15 mm Hg or more in SBP values between arms could identify patients at high risk of asymptomatic peripheral vascular disease and mortality [33]. The reduction in blood pressure values observed in this meta-analysis is probably due to the anti-hypertensive properties of magnesium, potassium, various vitamins and polyphenols contained in the fruit juice. However, we cannot perform a comprehensive and quantitative analysis to further evaluate the effect of these beneficial nutrients on serum cholesterol and blood pressure due to the limited RCT data. The causal conclusion remains to be evaluated under conditions of exact quantity of beneficial nutrients of fruit juice and high-quality RCTs with longer-term intervention duration.

Our study has several strengths. Firstly, the articles included in the meta-analysis are RCTs which have fewer methodological biases than observational studies such as case-control studies or cohort studies. Secondly, the relatively large number of pooled subjects provided the greater statistical power to examine a small intervention effect. Third, we used only one intervention group for each trial when calculating the pooled effect for studies with ≥2 intervention groups in order to avoid counting control patients more than once. In addition, no significant statistic heterogeneity was observed in the net change of TC, HDL-C, LDL-C, DBP, or SBP values. Moreover, the Egger’s tests showed no significant asymmetry of funnel plot for overall pooled effect of TC, HDL-C, LDL-C, DBP, or SBP values.

Potential limitations of the available data are inevitable. First, exact doses of vitamins contained in fruit juice in the 19 trials included in the current meta-analysis were unavailable. Additionally, the doses of total polyphenols in fruit juice ranged from 65.2 to 2660 mg/d (median: 927 mg/d) across the included studies. Although the wide range of total-polyphenols dose did not cause the significant heterogeneity of the pooled effects of TC, HDL-C, and LDL-C concentrations, it might have affected the overall outcome of the analyses. Second, the results of meta-regression analysis did not show significant dose-responsive effect between fruit juice and DBP (*P* for trend = 0.45), or between low-dose fruit juice and TC concentration (*P* for trend = 0.49). Therefore, it is difficult to assess the optimal dose for a dietary intervention program as part of a health policy aimed at improving public hypertension and cholesterol status. Third, the study duration was short (from 2 wk to 3 mo) and the quality of the trials varied from low to high, with only 7 of the 19 studies, found to be in high quality. Finally, we cannot perform subgroup and sensitivity analyses for DBP and SBP values due to the limited number of studies and the causal conclusion remains to be evaluated in future.

In conclusion, the consumption of fruit juice borderlinely lowered DBP, but no statistically significant overall effect on TC, HDL-C, LDL-C and SBP values was observed. Subgroup analyses for TC concentrations found that fruit juice significantly reduced of TC concentrations in low-median dose of total polyphenols group. In addition, our meta-analysis also suggested that fruit juice might have more favorable effects on TC, LDL-C and HDL-C regulation in abnormal cholesterol group than the healthy people.
